# Crystal structure of (*R*)-2′-benz­yloxy-[1,1′-binaphthalen]-2-yl tri­fluoro­methane­sulfonate

**DOI:** 10.1107/S1600536814019096

**Published:** 2014-09-10

**Authors:** Rui M. B. Carrilho, Mariette M. Pereira, Teresa M. R. Maria, M. Ermelinda S. Eusébio, V. H. Rodrigues

**Affiliations:** a Chemistry Department, University of Coimbra, P-3004-516 Coimbra, Portugal; b CEMDRX, Physics Department, University of Coimbra, P-3004-516 Coimbra, Portugal

**Keywords:** crystal structure, (R)-BINOL, binaphth­yl, sulfonate, chiral

## Abstract

In the title compound, C_28_H_19_F_3_O_4_S, a new 2′-benz­yloxy (*R*)-BINOL derivative containing a tri­fluoro­methane­sulfonate group in the 2-position, the planes of the two naphthyl ring systems (r.m.s. deviations = 0.012 and 0.019 Å) are at an angle of 73.36 (2)°, and the planes of the benzyl ring and the naphthyl ring system bound to the ether O atom are at an angle of 75.67 (4)°. In the crystal, mol­ecules are linked *via* C—H⋯F hydrogen bonds, forming chains propagating along [100]. The chains are linked *via* a weak C—F⋯π inter­action and weak π–π inter­actions [shortest inter-centroid distance = 3.9158 (12) Å], forming a three-dimensional structure. The absolute structure of the mol­ecule in the crystal was determined by resonant scattering [Flack parameter = 0.02 (6)].

## Related literature   

For the synthesis of some BINOL derivatives, see, for example: Carrilho *et al.* (2012 ▶, 2014 ▶). For the synthesis of related binaphthyl-based tri­fluoro­methane­sulfonate derivatives, see: Zeng *et al.* (2011 ▶); Singer & Buchwald (1999 ▶); Meškovà *et al.* (2011 ▶); Sälinger & Brückner (2009 ▶); Zheng *et al.* (2013 ▶). For the use of aryl tri­fluoro­methane­sulfonate derivatives as inter­mediates in Buchwald–Hartwig aminations, see: Louie *et al.* (1997 ▶); Ahman & Buchwald (1997 ▶); Meadows *et al.* (2008 ▶). For a review of the synthesis and catalytic applications of binaphthyl-based phosphine and phosphite ligands, see: Sakai *et al.* (1993 ▶); Yan & Zhang (2006 ▶); Pereira *et al.* (2013 ▶).
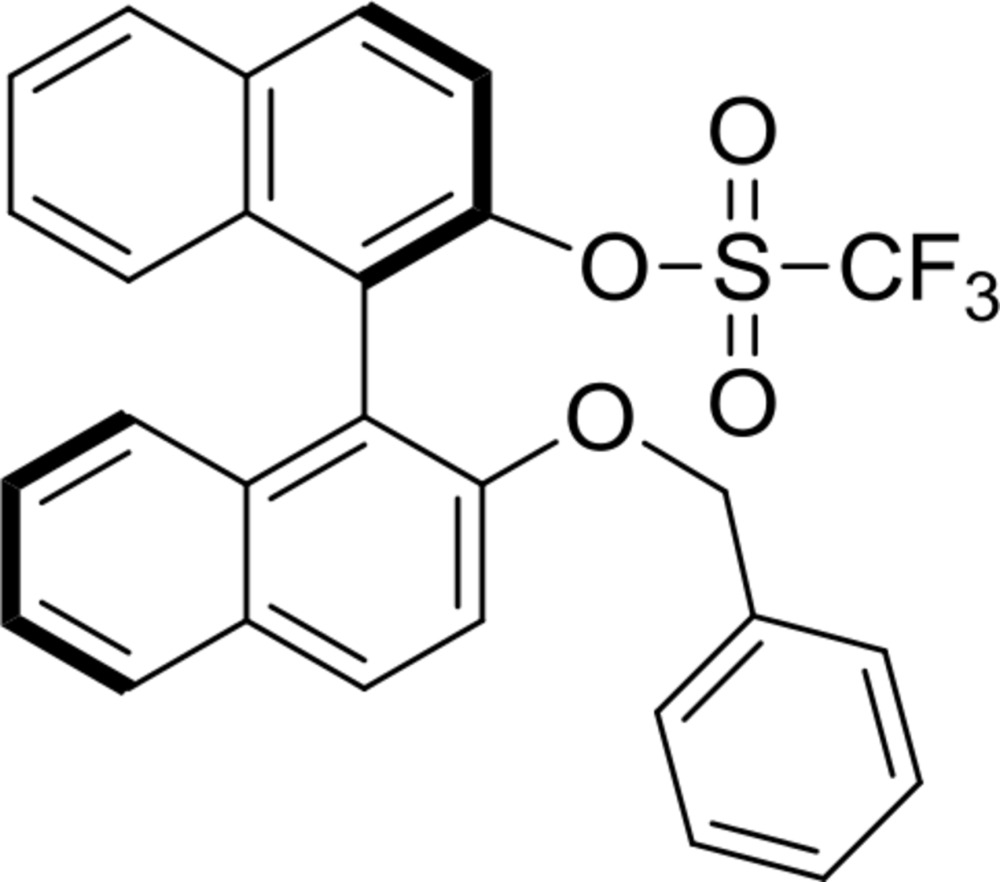


## Experimental   

### Crystal data  

C_28_H_19_F_3_O_4_S
*M* *_r_* = 508.49
Orthorhombic, 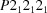

*a* = 9.3383 (4) Å
*b* = 12.3380 (5) Å
*c* = 20.5893 (8) Å
*V* = 2372.22 (17) Å^3^
*Z* = 4
Mo *K*α radiation
μ = 0.19 mm^−1^
*T* = 293 K
0.36 × 0.28 × 0.1 mm

### Data collection  

Bruker APEXII diffractometer
Absorption correction: multi-scan (*SADABS*; Bruker, 2004 ▶) *T*_min_ = 0.890, *T*_max_ = 1.000
42588 measured reflections
5367 independent reflections
4373 reflections with *I* > 2σ(*I*)
*R*_int_ = 0.038

### Refinement  

*R*[*F* ^2^ > 2σ(*F* ^2^)] = 0.037
*wR*(*F* ^2^) = 0.088
*S* = 1.06
5367 reflections
326 parameters
H-atom parameters constrained
Δρ_max_ = 0.18 e Å^−3^
Δρ_min_ = −0.24 e Å^−3^
Absolute structure: Flack (1983 ▶)
Absolute structure parameter: 0.02 (6)

### 

Data collection: *APEX2* (Bruker, 2004 ▶); cell refinement: *SAINT* (Bruker, 2004 ▶); data reduction: *SAINT*; program(s) used to solve structure: *SHELXS97* (Sheldrick, 2008 ▶); program(s) used to refine structure: *SHELXL97* (Sheldrick, 2008 ▶); molecular graphics: *ORTEPII* (Johnson, 1976 ▶) and *Mercury* (Macrae *et al.* 2008 ▶); software used to prepare material for publication: *SHELXL97* and *PLATON* (Spek, 2009 ▶).

## Supplementary Material

Crystal structure: contains datablock(s) I, global. DOI: 10.1107/S1600536814019096/su2775sup1.cif

Structure factors: contains datablock(s) I. DOI: 10.1107/S1600536814019096/su2775Isup2.hkl

**Fig. 1.**
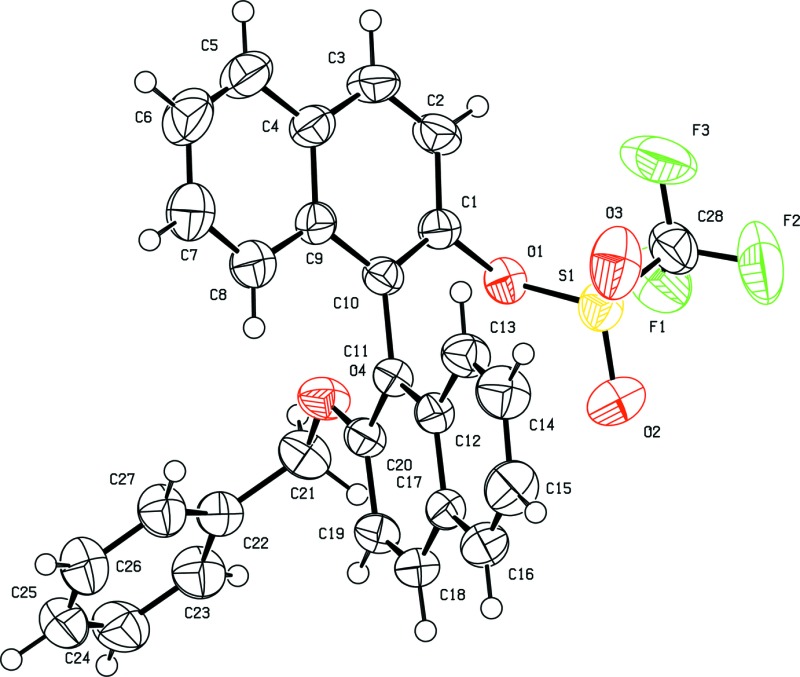
A view of the molecular structure of the title molecule, with atom labelling. Displacement ellipsoids are drawn at the 50% level.

. DOI: 10.1107/S1600536814019096/su2775fig1.tif
A view of the mol­ecular structure of the title mol­ecule, with atom labelling. Displacement ellipsoids are drawn at the 50% level.

**Fig. 2.**
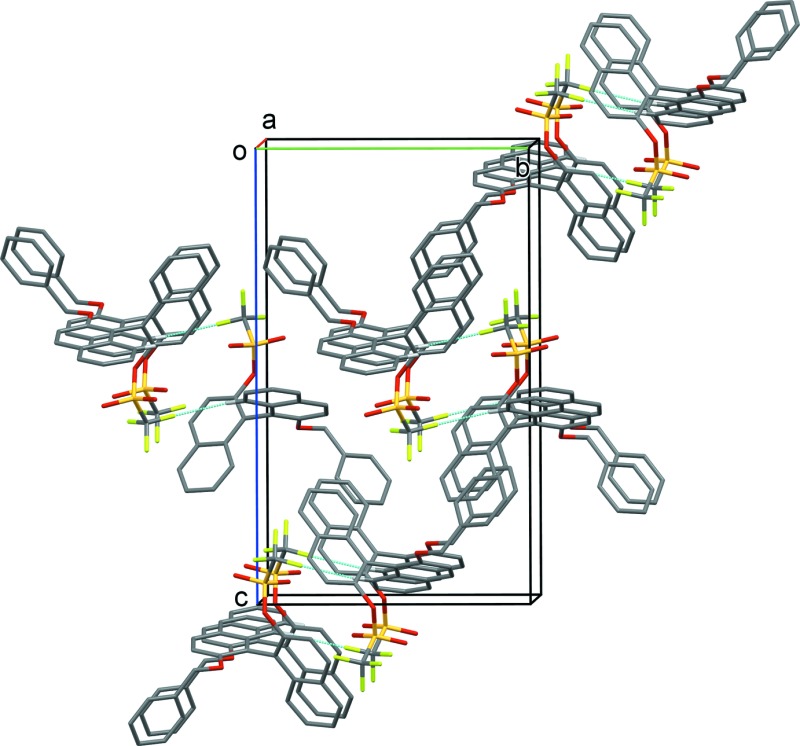
A view along the a axis of the crystal packing of the title compound. The C-H···F hydrogen bonds are shown as dashed lines (see Table 1 for details).

. DOI: 10.1107/S1600536814019096/su2775fig2.tif
A view along the a axis of the crystal packing of the title compound. The C-H⋯F hydrogen bonds are shown as dashed lines (see Table 1 for details).

CCDC reference: 1020770

Additional supporting information:  crystallographic information; 3D view; checkCIF report

## Figures and Tables

**Table 1:** Hydrogen-bond geometry (Å, °) *Cg*1 is the centroid of the C1–C4/C9/C10 ring

*D*—H⋯*A*	*D*—H	H⋯*A*	*D*⋯*A*	*D*—H⋯*A*
C13—H13⋯F3^i^	0.93	2.50	3.357 (2)	153
C28—F3⋯*Cg*1^ii^	1.29 (1)	3.61 (1)	4.632 (3)	136 (1)

Symmetry codes: (i) 
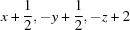
; (ii) 
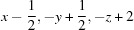
.

## supplementary crystallographic information

### S1. Experimental

The title compound was synthesized from (*R*)-BINOL according to an optimized two step procedure. To a solution of (*R*)-BINOL (5.00 g, 17 mmol), dried azeotropically with toluene, triphenylphosphine (PPh3) (4.5 g, 17 mmol) and benzyl alcohol (2.1 ml, 20 mmol), in dry THF (100 ml), diethyl azodicarboxylate (DEAD) (40% in toluene, 7.5 ml, 17 mmol) was added drop wise, at 273 K and the mixture was stirred at 298 K, for 48 h. After quenching with water, the solvent was evaporated under reduced pressure and the crude mixture was dissolved in dichloromethane (50 ml). The organic layer was washed with brine (3 × 50 ml) and water (3 × 50 ml) and dried over anhydrous Na_2_SO_4_. After removal of the solvent under reduced pressure, the product was isolated by column chromatography on silica gel, using a mixture of CH_2_Cl_2_/n-hexane (2:1) as eluent, and was further purified by recrystallization from toluene/n-hexane yielding white crystals. The intermediate product, (*R*)-2'-(benzyloxy)-1,1'-binaphthyl-2-ol (L), with a white crystalline aspect, was obtained in 91% yield (5.85 g, 15.5 mmol). The respective NMR data obtained are in agreement with published values [Takahashi, M. & Ogasawara, K. (1997). *Tetrahedron Asymmetry*, 8, 3125–3130]. Next, to a solution of (L) (2.82 g, 7.5 mmol) in anhydrous CH_2_Cl_2_ (15 ml), were added sequentially and drop wise, at 273 K under a nitrogen atmosphere, pyridine (1.0 ml, 12 mmol) and trifluoromethanesulfonic anhydride (triflic anhydride) (1.5 ml, 9 mmol.) The mixture, which produced a red solution, was allowed to warm to room temperature and stirred for 6 h. n-Hexane (20 ml) was then added, and the mixture was passed over a silica gel column (previously activated at 473 K). The silica gel column was washed with 40 ml of a mixture of CH_2_Cl_2_/n-hexane (1:1). After removal of the solvents under reduced pressure, the title compound was obtained as a white solid in 87% yield (3.30 g, 6.5 mmol). Crystals suitable for X-ray diffraction analysis were obtained after dissolution of the title compound (5 mg ml^-1^) in ethyl acetate, and left for the solvent to evaporate in air at room temperature for 48 h.

Spectroscopic data for the title compound: ^1^H NMR (CDCl_3_, TMS, 400 MHz) δ (p.p.m.) 5.01 (s, 2H, OCH_2_Ph), 6.92–6.99 (m, 3H, ArH), 7.03–7.05 (m, 3H, ArH), 7.13 (t, J=7.4 Hz, 1H, ArH), 7.19–7.27 (m, 3H, ArH), 7.29 (d, J=9.2 Hz, 1H, ArH), 7.36–7.40 (m, 1H, ArH), 7.46 (d, J=8.8 Hz, 1H, ArH), 7.73 (d,J=8.0 Hz, 1H, ArH), 7.81–7.86 (m, 2H, ArH), 7.89 (d, J=8.8 Hz, 1H, ArH). ^13^C NMR (CDCl_3_, TMS, 100 MHz) δ (p.p.m.) 70.8 (OCH_2_Ph), 114.7 (ArC), 116.2 (ArC), 116.8 (CF_3_), 119.7 (ArC), 120.0 (ArC), 124.0 (ArC), 125.2 (ArC), 126.7 (ArC), 126.9 (ArC), 127.0 (ArC), 127.1 (ArC), 127.5 (ArC), 127.5 (ArC), 127.6 (ArC), 128.2 (ArC), 128.3 (ArC), 128.4 (ArC), 129.1 (ArC), 130.4 (ArC), 131.1 (ArC), 132.7 (ArC), 133.8 (ArC), 133.8 (ArC), 137.3 (OCH_2_C_Ph_), 145.8 (COTf), 154.4 (COBn). ^19^F NMR (CDCl_3_, TFA, 376 MHz) δ (p.p.m.) -73.61 (OS(O)2CF_3_). Mp: 128–131 °C.

### S2. Refinement

All the H atoms were placed in idealized positions and refined as riding atoms: C—H = 0.93 - 0.97 Å with *U*_iso_(H) = 1.2*U*_eq_(C)].

### Crystal data

**Table:**

C_28_H_19_F_3_O_4_S	*F*(000) = 1048
*M**_r_* = 508.49	*D*_x_ = 1.424 Mg m^−^^3^
Orthorhombic, *P*2_1_2_1_2_1_	Mo *K*α radiation, λ = 0.71073 Å
Hall symbol: P 2ac 2ab	Cell parameters from 6182 reflections
*a* = 9.3383 (4) Å	θ = 2.6–22.2°
*b* = 12.3380 (5) Å	µ = 0.19 mm^−^^1^
*c* = 20.5893 (8) Å	*T* = 293 K
*V* = 2372.22 (17) Å^3^	Prismatic, colourless
*Z* = 4	0.36 × 0.28 × 0.1 mm

### Data collection

**Table:**

Bruker APEXII diffractometer	5367 independent reflections
Radiation source: fine-focus sealed tube	4373 reflections with *I* > 2σ(*I*)
Graphite monochromator	*R*_int_ = 0.038
φ and ω scans	θ_max_ = 27.5°, θ_min_ = 1.9°
Absorption correction: multi-scan (*SADABS*; Bruker, 2004)	*h* = −11→12
*T*_min_ = 0.890, *T*_max_ = 1.000	*k* = −15→15
42588 measured reflections	*l* = −26→26

### Refinement

**Table:**

Refinement on *F*^2^	Hydrogen site location: inferred from neighbouring sites
Least-squares matrix: full	H-atom parameters constrained
*R*[*F*^2^ > 2σ(*F*^2^)] = 0.037	*w* = 1/[σ^2^(*F*_o_^2^) + (0.0437*P*)^2^ + 0.1954*P*] where *P* = (*F*_o_^2^ + 2*F*_c_^2^)/3
*wR*(*F*^2^) = 0.088	(Δ/σ)_max_ = 0.001
*S* = 1.06	Δρ_max_ = 0.18 e Å^−^^3^
5367 reflections	Δρ_min_ = −0.24 e Å^−^^3^
326 parameters	Extinction correction: *SHELXL97* (Sheldrick, 2008), Fc^*^=kFc[1+0.001xFc^2^λ^3^/sin(2θ)]^-1/4^
0 restraints	Extinction coefficient: 0.0058 (7)
Primary atom site location: structure-invariant direct methods	Absolute structure: Flack (1983)
Secondary atom site location: difference Fourier map	Absolute structure parameter: 0.02 (6)

### Special details

**Table:**

Geometry. All e.s.d.'s (except the e.s.d. in the dihedral angle between two l.s. planes) are estimated using the full covariance matrix. The cell e.s.d.'s are taken into account individually in the estimation of e.s.d.'s in distances, angles and torsion angles; correlations between e.s.d.'s in cell parameters are only used when they are defined by crystal symmetry. An approximate (isotropic) treatment of cell e.s.d.'s is used for estimating e.s.d.'s involving l.s. planes.
Refinement. Refinement of *F*^2^ against ALL reflections. The weighted *R*-factor *wR* and goodness of fit *S* are based on *F*^2^, conventional *R*-factors *R* are based on *F*, with *F* set to zero for negative *F*^2^. The threshold expression of *F*^2^ > σ(*F*^2^) is used only for calculating *R*-factors(gt) *etc*. and is not relevant to the choice of reflections for refinement. *R*-factors based on *F*^2^ are statistically about twice as large as those based on *F*, and *R*- factors based on ALL data will be even larger.

### Fractional atomic coordinates and isotropic or equivalent isotropic displacement parameters (Å^2^)

**Table:**

	*x*	*y*	*z*	*U*_iso_*/*U*_eq_	
S1	0.71125 (5)	0.03924 (4)	0.94675 (2)	0.04816 (14)	
O1	0.62251 (12)	0.04090 (10)	1.01056 (5)	0.0417 (3)	
O2	0.74153 (18)	−0.06979 (12)	0.93075 (8)	0.0706 (4)	
O3	0.81614 (18)	0.12109 (14)	0.94647 (8)	0.0759 (5)	
C28	0.5709 (3)	0.08085 (18)	0.89039 (11)	0.0641 (6)	
F1	0.46451 (16)	0.01259 (12)	0.89093 (7)	0.0829 (4)	
F2	0.6254 (2)	0.08254 (15)	0.83119 (7)	0.1095 (6)	
F3	0.5251 (2)	0.17688 (12)	0.90464 (9)	0.1210 (8)	
C1	0.66233 (18)	0.11054 (13)	1.06373 (8)	0.0381 (4)	
C2	0.5916 (2)	0.20964 (15)	1.06794 (9)	0.0471 (5)	
H2	0.5246	0.2301	1.0368	0.056*	
C3	0.6231 (2)	0.27570 (16)	1.11882 (10)	0.0533 (5)	
H3	0.5767	0.3421	1.1227	0.064*	
C4	0.7249 (2)	0.24510 (14)	1.16576 (9)	0.0462 (4)	
C5	0.7608 (3)	0.31324 (17)	1.21878 (11)	0.0633 (6)	
H5	0.7165	0.3804	1.2229	0.076*	
C6	0.8584 (3)	0.2820 (2)	1.26350 (11)	0.0705 (7)	
H6	0.8812	0.3281	1.2977	0.085*	
C7	0.9252 (3)	0.18142 (19)	1.25880 (10)	0.0664 (6)	
H7	0.9915	0.1607	1.2901	0.080*	
C8	0.8943 (2)	0.11300 (17)	1.20867 (9)	0.0520 (5)	
H8	0.9400	0.0462	1.2061	0.062*	
C9	0.7933 (2)	0.14259 (14)	1.16045 (8)	0.0405 (4)	
C10	0.76185 (18)	0.07382 (13)	1.10677 (8)	0.0362 (4)	
C11	0.83484 (18)	−0.03262 (14)	1.09688 (8)	0.0361 (4)	
C12	0.97941 (18)	−0.03635 (14)	1.07440 (8)	0.0368 (4)	
C13	1.0607 (2)	0.05821 (15)	1.06183 (9)	0.0473 (4)	
H13	1.0190	0.1260	1.0674	0.057*	
C14	1.1994 (2)	0.05104 (18)	1.04172 (10)	0.0588 (5)	
H14	1.2512	0.1140	1.0337	0.071*	
C15	1.2648 (2)	−0.05005 (19)	1.03296 (10)	0.0604 (5)	
H15	1.3602	−0.0538	1.0203	0.072*	
C16	1.1901 (2)	−0.14184 (17)	1.04285 (9)	0.0500 (5)	
H16	1.2341	−0.2085	1.0361	0.060*	
C17	1.04466 (19)	−0.13834 (14)	1.06346 (8)	0.0396 (4)	
C18	0.9652 (2)	−0.23321 (15)	1.07409 (9)	0.0447 (4)	
H18	1.0073	−0.3002	1.0661	0.054*	
C19	0.8274 (2)	−0.22913 (14)	1.09589 (9)	0.0438 (4)	
H19	0.7767	−0.2929	1.1031	0.053*	
C20	0.76214 (18)	−0.12812 (13)	1.10741 (8)	0.0374 (4)	
O4	0.62534 (13)	−0.11801 (10)	1.13046 (7)	0.0486 (3)	
C21	0.5362 (2)	−0.21227 (16)	1.13402 (10)	0.0506 (5)	
H21A	0.5492	−0.2543	1.0946	0.061*	
H21B	0.4370	−0.1892	1.1354	0.061*	
C22	0.56468 (19)	−0.28464 (15)	1.19158 (9)	0.0432 (4)	
C23	0.4871 (2)	−0.37900 (17)	1.19706 (11)	0.0568 (5)	
H23	0.4214	−0.3971	1.1649	0.068*	
C24	0.5055 (3)	−0.44718 (18)	1.24960 (12)	0.0673 (6)	
H24	0.4516	−0.5103	1.2529	0.081*	
C25	0.6022 (3)	−0.42192 (19)	1.29639 (11)	0.0657 (6)	
H25	0.6146	−0.4678	1.3318	0.079*	
C26	0.6814 (3)	−0.32918 (19)	1.29156 (11)	0.0654 (6)	
H26	0.7486	−0.3125	1.3233	0.079*	
C27	0.6614 (2)	−0.25998 (17)	1.23930 (10)	0.0543 (5)	
H27	0.7141	−0.1962	1.2366	0.065*	

### Atomic displacement parameters (Å^2^)

**Table:**

	*U*^11^	*U*^22^	*U*^33^	*U*^12^	*U*^13^	*U*^23^
S1	0.0493 (3)	0.0501 (3)	0.0450 (2)	−0.0092 (2)	0.0044 (2)	−0.0009 (2)
O1	0.0400 (6)	0.0445 (7)	0.0407 (6)	−0.0051 (6)	−0.0015 (5)	0.0028 (6)
O2	0.0793 (10)	0.0590 (9)	0.0736 (10)	0.0117 (8)	0.0072 (8)	−0.0157 (7)
O3	0.0716 (10)	0.0955 (12)	0.0607 (9)	−0.0437 (9)	0.0134 (8)	−0.0032 (8)
C28	0.0933 (17)	0.0476 (12)	0.0513 (12)	−0.0119 (13)	−0.0123 (12)	0.0061 (10)
F1	0.0779 (9)	0.0843 (10)	0.0867 (9)	−0.0169 (8)	−0.0295 (8)	0.0098 (8)
F2	0.1557 (16)	0.1263 (13)	0.0465 (8)	−0.0335 (13)	−0.0093 (9)	0.0187 (8)
F3	0.193 (2)	0.0576 (9)	0.1125 (13)	0.0383 (11)	−0.0741 (14)	−0.0055 (8)
C1	0.0384 (9)	0.0354 (9)	0.0407 (9)	0.0003 (7)	0.0022 (7)	−0.0006 (7)
C2	0.0448 (10)	0.0429 (10)	0.0536 (11)	0.0117 (9)	−0.0018 (9)	0.0086 (9)
C3	0.0568 (12)	0.0395 (11)	0.0636 (13)	0.0160 (9)	0.0064 (10)	−0.0011 (10)
C4	0.0508 (11)	0.0397 (10)	0.0483 (10)	0.0018 (9)	0.0113 (9)	−0.0030 (8)
C5	0.0801 (16)	0.0489 (12)	0.0609 (13)	0.0017 (11)	0.0130 (12)	−0.0143 (10)
C6	0.0911 (18)	0.0670 (15)	0.0535 (13)	−0.0082 (13)	0.0002 (13)	−0.0184 (11)
C7	0.0761 (16)	0.0744 (15)	0.0486 (12)	0.0002 (13)	−0.0097 (11)	−0.0050 (11)
C8	0.0565 (12)	0.0530 (12)	0.0463 (11)	0.0045 (10)	−0.0041 (9)	−0.0009 (9)
C9	0.0447 (10)	0.0388 (9)	0.0380 (9)	−0.0007 (9)	0.0049 (8)	0.0005 (7)
C10	0.0365 (9)	0.0327 (9)	0.0393 (9)	0.0019 (7)	0.0046 (7)	0.0041 (7)
C11	0.0399 (9)	0.0331 (9)	0.0353 (8)	0.0036 (8)	−0.0011 (7)	0.0023 (7)
C12	0.0389 (9)	0.0370 (9)	0.0345 (8)	0.0014 (8)	−0.0021 (7)	0.0011 (7)
C13	0.0462 (10)	0.0406 (10)	0.0552 (11)	−0.0012 (8)	0.0039 (9)	0.0038 (9)
C14	0.0498 (11)	0.0572 (13)	0.0693 (13)	−0.0108 (11)	0.0071 (10)	0.0057 (10)
C15	0.0407 (10)	0.0726 (15)	0.0679 (13)	0.0028 (11)	0.0122 (9)	0.0018 (11)
C16	0.0450 (11)	0.0527 (12)	0.0524 (11)	0.0110 (9)	0.0071 (9)	−0.0003 (9)
C17	0.0437 (10)	0.0406 (10)	0.0344 (9)	0.0073 (8)	−0.0009 (7)	0.0004 (7)
C18	0.0529 (11)	0.0366 (10)	0.0444 (10)	0.0082 (9)	0.0008 (8)	−0.0018 (8)
C19	0.0525 (11)	0.0326 (10)	0.0463 (10)	−0.0011 (8)	0.0007 (8)	0.0026 (8)
C20	0.0372 (9)	0.0352 (9)	0.0398 (9)	0.0027 (7)	0.0010 (7)	0.0048 (7)
O4	0.0422 (7)	0.0387 (7)	0.0651 (8)	−0.0009 (6)	0.0113 (6)	0.0073 (6)
C21	0.0425 (10)	0.0474 (11)	0.0618 (12)	−0.0080 (9)	−0.0027 (9)	0.0084 (9)
C22	0.0368 (10)	0.0425 (10)	0.0502 (10)	−0.0028 (8)	0.0053 (8)	0.0012 (8)
C23	0.0567 (13)	0.0494 (12)	0.0643 (13)	−0.0165 (10)	0.0002 (10)	0.0014 (10)
C24	0.0769 (15)	0.0464 (12)	0.0785 (15)	−0.0107 (12)	0.0119 (13)	0.0092 (12)
C25	0.0802 (16)	0.0589 (14)	0.0580 (13)	0.0100 (13)	0.0094 (12)	0.0137 (11)
C26	0.0714 (15)	0.0728 (15)	0.0520 (12)	−0.0009 (13)	−0.0048 (11)	0.0065 (11)
C27	0.0580 (12)	0.0529 (12)	0.0521 (11)	−0.0130 (10)	−0.0019 (10)	0.0029 (9)

### Geometric parameters (Å, º)

**Table:**

S1—O3	1.4069 (15)	C13—C14	1.362 (3)
S1—O2	1.4136 (15)	C13—H13	0.9300
S1—O1	1.5535 (12)	C14—C15	1.400 (3)
S1—C28	1.824 (2)	C14—H14	0.9300
O1—C1	1.440 (2)	C15—C16	1.346 (3)
C28—F3	1.293 (3)	C15—H15	0.9300
C28—F1	1.302 (3)	C16—C17	1.423 (3)
C28—F2	1.321 (3)	C16—H16	0.9300
C1—C10	1.362 (2)	C17—C18	1.403 (3)
C1—C2	1.393 (2)	C18—C19	1.363 (3)
C2—C3	1.360 (3)	C18—H18	0.9300
C2—H2	0.9300	C19—C20	1.407 (2)
C3—C4	1.407 (3)	C19—H19	0.9300
C3—H3	0.9300	C20—O4	1.368 (2)
C4—C5	1.418 (3)	O4—C21	1.432 (2)
C4—C9	1.421 (2)	C21—C22	1.508 (3)
C5—C6	1.352 (3)	C21—H21A	0.9700
C5—H5	0.9300	C21—H21B	0.9700
C6—C7	1.392 (3)	C22—C27	1.369 (3)
C6—H6	0.9300	C22—C23	1.376 (3)
C7—C8	1.364 (3)	C23—C24	1.381 (3)
C7—H7	0.9300	C23—H23	0.9300
C8—C9	1.417 (3)	C24—C25	1.357 (3)
C8—H8	0.9300	C24—H24	0.9300
C9—C10	1.424 (2)	C25—C26	1.366 (3)
C10—C11	1.493 (2)	C25—H25	0.9300
C11—C20	1.377 (2)	C26—C27	1.386 (3)
C11—C12	1.428 (2)	C26—H26	0.9300
C12—C13	1.416 (3)	C27—H27	0.9300
C12—C17	1.416 (2)		
			
O3—S1—O2	122.88 (11)	C14—C13—C12	120.78 (18)
O3—S1—O1	111.42 (8)	C14—C13—H13	119.6
O2—S1—O1	108.45 (9)	C12—C13—H13	119.6
O3—S1—C28	107.20 (11)	C13—C14—C15	120.76 (19)
O2—S1—C28	105.27 (11)	C13—C14—H14	119.6
O1—S1—C28	98.69 (10)	C15—C14—H14	119.6
C1—O1—S1	120.87 (10)	C16—C15—C14	120.26 (17)
F3—C28—F1	109.8 (2)	C16—C15—H15	119.9
F3—C28—F2	108.80 (19)	C14—C15—H15	119.9
F1—C28—F2	108.19 (19)	C15—C16—C17	120.95 (18)
F3—C28—S1	110.54 (16)	C15—C16—H16	119.5
F1—C28—S1	111.13 (14)	C17—C16—H16	119.5
F2—C28—S1	108.3 (2)	C18—C17—C12	119.26 (16)
C10—C1—C2	125.14 (16)	C18—C17—C16	121.73 (16)
C10—C1—O1	118.18 (14)	C12—C17—C16	119.00 (17)
C2—C1—O1	116.65 (15)	C19—C18—C17	121.32 (17)
C3—C2—C1	118.13 (17)	C19—C18—H18	119.3
C3—C2—H2	120.9	C17—C18—H18	119.3
C1—C2—H2	120.9	C18—C19—C20	119.78 (17)
C2—C3—C4	120.98 (17)	C18—C19—H19	120.1
C2—C3—H3	119.5	C20—C19—H19	120.1
C4—C3—H3	119.5	O4—C20—C11	115.90 (14)
C3—C4—C5	121.96 (18)	O4—C20—C19	122.91 (15)
C3—C4—C9	119.32 (17)	C11—C20—C19	121.18 (16)
C5—C4—C9	118.71 (19)	C20—O4—C21	119.09 (14)
C6—C5—C4	121.0 (2)	O4—C21—C22	114.75 (16)
C6—C5—H5	119.5	O4—C21—H21A	108.6
C4—C5—H5	119.5	C22—C21—H21A	108.6
C5—C6—C7	120.6 (2)	O4—C21—H21B	108.6
C5—C6—H6	119.7	C22—C21—H21B	108.6
C7—C6—H6	119.7	H21A—C21—H21B	107.6
C8—C7—C6	120.6 (2)	C27—C22—C23	118.46 (18)
C8—C7—H7	119.7	C27—C22—C21	123.30 (17)
C6—C7—H7	119.7	C23—C22—C21	118.22 (17)
C7—C8—C9	120.77 (19)	C22—C23—C24	121.0 (2)
C7—C8—H8	119.6	C22—C23—H23	119.5
C9—C8—H8	119.6	C24—C23—H23	119.5
C8—C9—C4	118.34 (16)	C25—C24—C23	119.9 (2)
C8—C9—C10	121.85 (16)	C25—C24—H24	120.0
C4—C9—C10	119.80 (16)	C23—C24—H24	120.0
C1—C10—C9	116.60 (15)	C24—C25—C26	120.1 (2)
C1—C10—C11	121.02 (15)	C24—C25—H25	120.0
C9—C10—C11	122.37 (15)	C26—C25—H25	120.0
C20—C11—C12	119.31 (16)	C25—C26—C27	120.0 (2)
C20—C11—C10	120.39 (14)	C25—C26—H26	120.0
C12—C11—C10	120.27 (16)	C27—C26—H26	120.0
C13—C12—C17	118.19 (15)	C22—C27—C26	120.6 (2)
C13—C12—C11	122.66 (16)	C22—C27—H27	119.7
C17—C12—C11	119.15 (16)	C26—C27—H27	119.7
			
O3—S1—O1—C1	−7.53 (15)	C1—C10—C11—C12	104.50 (19)
O2—S1—O1—C1	130.68 (13)	C9—C10—C11—C12	−74.6 (2)
C28—S1—O1—C1	−119.94 (13)	C20—C11—C12—C13	179.35 (16)
O3—S1—C28—F3	−53.6 (2)	C10—C11—C12—C13	1.3 (2)
O2—S1—C28—F3	174.03 (18)	C20—C11—C12—C17	0.0 (2)
O1—S1—C28—F3	62.1 (2)	C10—C11—C12—C17	−178.01 (14)
O3—S1—C28—F1	−175.81 (17)	C17—C12—C13—C14	−2.0 (3)
O2—S1—C28—F1	51.9 (2)	C11—C12—C13—C14	178.62 (18)
O1—S1—C28—F1	−60.08 (18)	C12—C13—C14—C15	0.0 (3)
O3—S1—C28—F2	65.48 (18)	C13—C14—C15—C16	1.6 (3)
O2—S1—C28—F2	−66.86 (17)	C14—C15—C16—C17	−1.2 (3)
O1—S1—C28—F2	−178.79 (15)	C13—C12—C17—C18	−178.51 (17)
S1—O1—C1—C10	−85.42 (17)	C11—C12—C17—C18	0.9 (2)
S1—O1—C1—C2	96.53 (17)	C13—C12—C17—C16	2.4 (2)
C10—C1—C2—C3	−0.1 (3)	C11—C12—C17—C16	−178.21 (16)
O1—C1—C2—C3	177.79 (17)	C15—C16—C17—C18	−179.92 (19)
C1—C2—C3—C4	0.3 (3)	C15—C16—C17—C12	−0.9 (3)
C2—C3—C4—C5	179.2 (2)	C12—C17—C18—C19	−1.2 (3)
C2—C3—C4—C9	−1.3 (3)	C16—C17—C18—C19	177.82 (18)
C3—C4—C5—C6	179.7 (2)	C17—C18—C19—C20	0.7 (3)
C9—C4—C5—C6	0.2 (3)	C12—C11—C20—O4	178.43 (15)
C4—C5—C6—C7	−0.6 (4)	C10—C11—C20—O4	−3.6 (2)
C5—C6—C7—C8	0.6 (4)	C12—C11—C20—C19	−0.5 (2)
C6—C7—C8—C9	−0.2 (4)	C10—C11—C20—C19	177.46 (16)
C7—C8—C9—C4	−0.2 (3)	C18—C19—C20—O4	−178.70 (16)
C7—C8—C9—C10	178.4 (2)	C18—C19—C20—C11	0.2 (3)
C3—C4—C9—C8	−179.31 (18)	C11—C20—O4—C21	171.35 (15)
C5—C4—C9—C8	0.2 (3)	C19—C20—O4—C21	−9.7 (2)
C3—C4—C9—C10	2.1 (3)	C20—O4—C21—C22	79.1 (2)
C5—C4—C9—C10	−178.42 (17)	O4—C21—C22—C27	3.9 (3)
C2—C1—C10—C9	0.9 (3)	O4—C21—C22—C23	−177.56 (17)
O1—C1—C10—C9	−177.00 (14)	C27—C22—C23—C24	0.4 (3)
C2—C1—C10—C11	−178.24 (16)	C21—C22—C23—C24	−178.2 (2)
O1—C1—C10—C11	3.9 (2)	C22—C23—C24—C25	−0.7 (4)
C8—C9—C10—C1	179.60 (17)	C23—C24—C25—C26	0.0 (4)
C4—C9—C10—C1	−1.8 (2)	C24—C25—C26—C27	0.9 (4)
C8—C9—C10—C11	−1.3 (3)	C23—C22—C27—C26	0.6 (3)
C4—C9—C10—C11	177.27 (16)	C21—C22—C27—C26	179.1 (2)
C1—C10—C11—C20	−73.5 (2)	C25—C26—C27—C22	−1.3 (3)
C9—C10—C11—C20	107.46 (19)		

### Hydrogen-bond geometry (Å, º)

Cg1 is the centroid of the C1-C4/C9/C10 ring

**Table:**

*D*—H···*A*	*D*—H	H···*A*	*D*···*A*	*D*—H···*A*
C13—H13···F3^i^	0.93	2.50	3.357 (2)	153
C28—F3···*Cg*1^ii^	1.29 (1)	3.61 (1)	4.632 (3)	136 (1)

Symmetry codes: (i) *x*+1/2, −*y*+1/2, −*z*+2; (ii) *x*−1/2, −*y*+1/2, −*z*+2.
