# Supplementary figures and images for: Crystal structure of (R)-2′-benz­yloxy-[1,1′-binaphthalen]-2-yl tri­fluoro­methane­sulfonate
Source: Acta Crystallogr Sect E Struct Rep Online. 2014 Sep 10;70(Pt 10):o1096–7. doi: 10.1107/S1600536814019096 (PMC4257171; doi:10.1107/S1600536814019096)

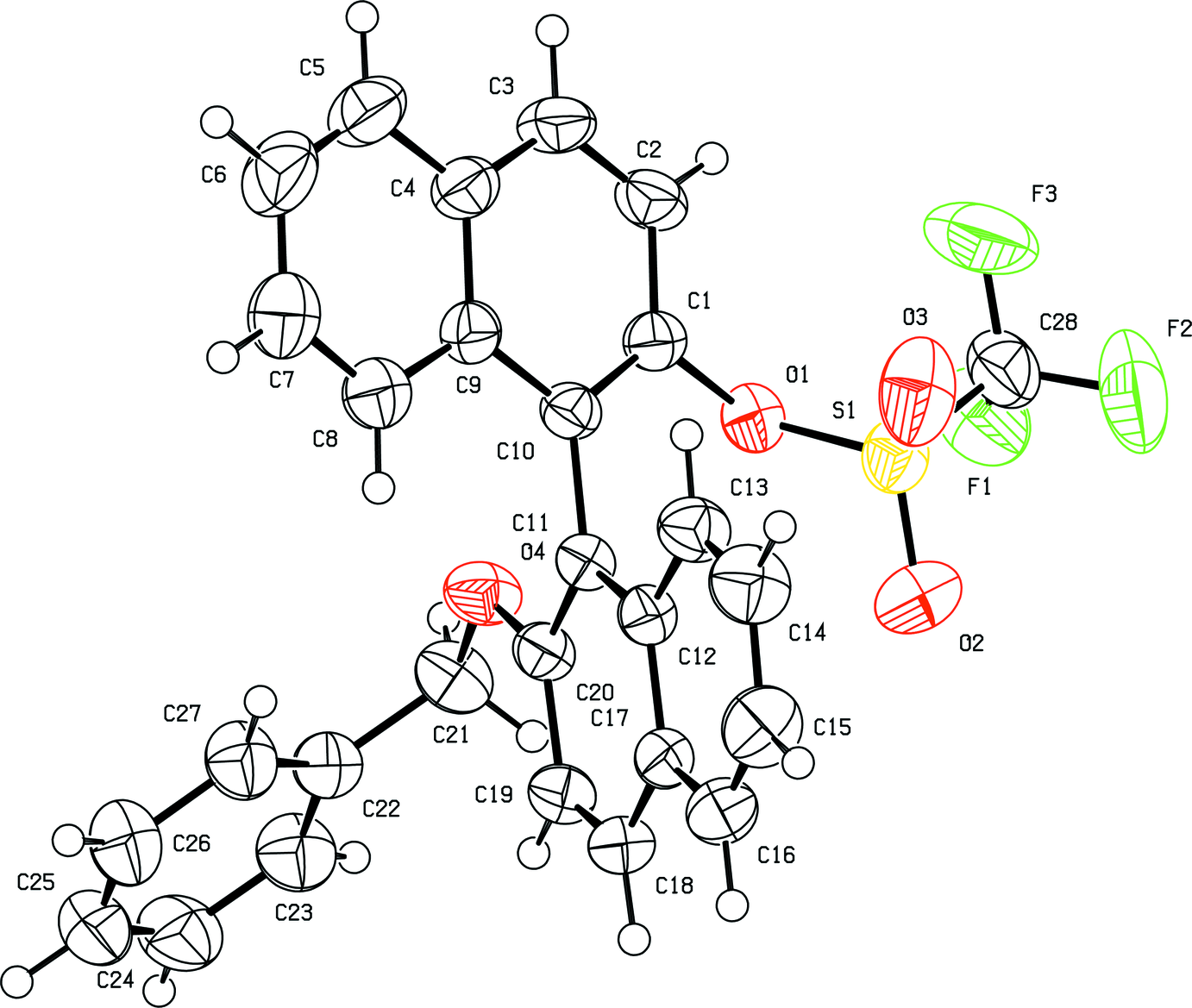

Supplement: Supplementary file 3 [file e-70-o1096-fig1.tif]

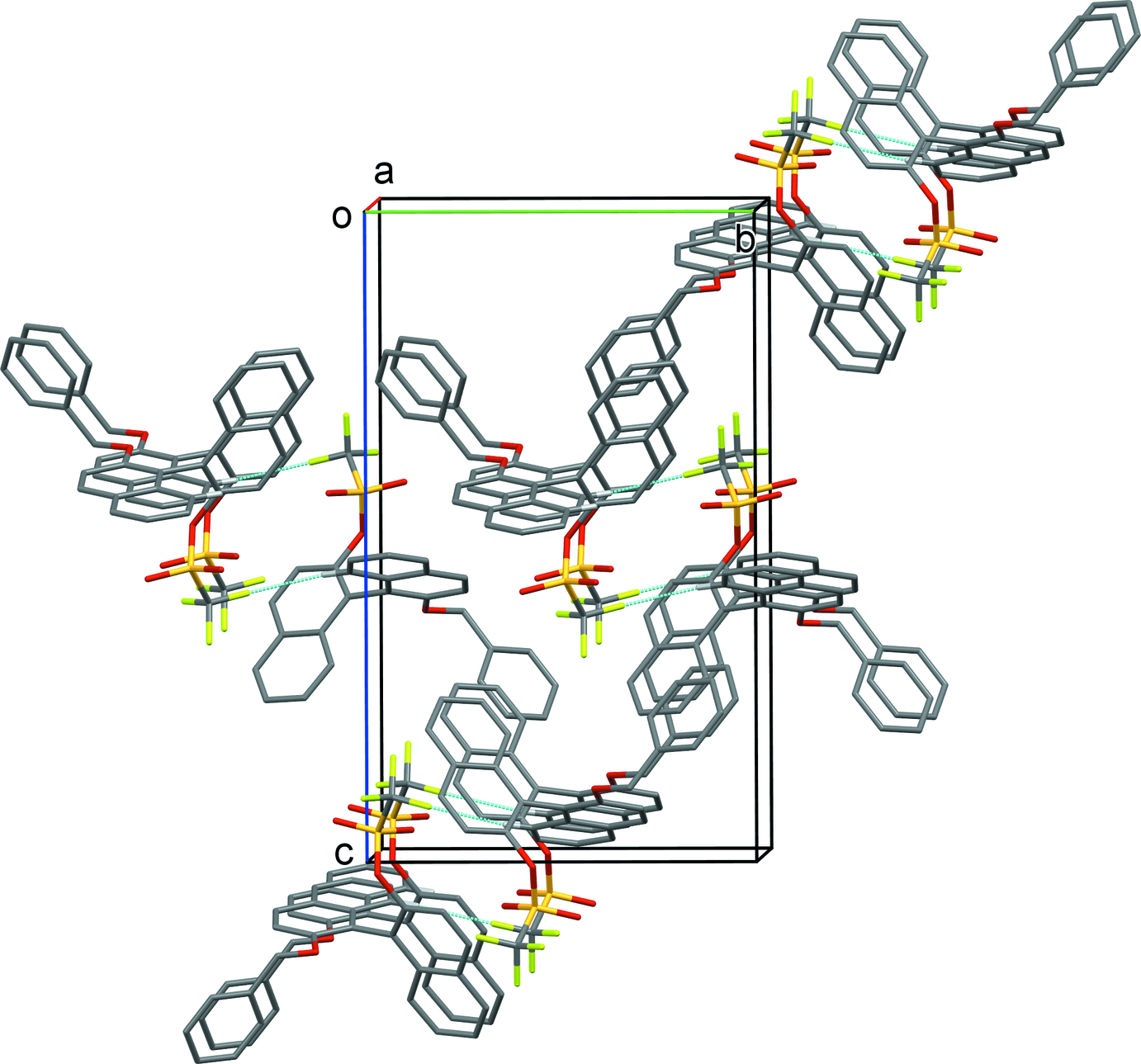

Supplement: Supplementary file 4 [file e-70-o1096-fig2.tif]
